# Impact of the Metal Center and Leaving Group on the Anticancer Activity of Organometallic Complexes of Pyridine-2-carbothioamide †

**DOI:** 10.3390/molecules26040833

**Published:** 2021-02-05

**Authors:** Jahanzaib Arshad, Kelvin K. H. Tong, Sanam Movassaghi, Tilo Söhnel, Stephen M. F. Jamieson, Muhammad Hanif, Christian G. Hartinger

**Affiliations:** 1 School of Chemical Sciences, University of Auckland, Private Bag 92019, Auckland 1142, New Zealand; zaibo.47@gmail.com (J.A.); kton030@aucklanduni.ac.nz (K.K.H.T.); smov524@aucklanduni.ac.nz (S.M.); t.soehnel@auckland.ac.nz (T.S.); 2 Maurice Wilkins Centre, University of Auckland, Private Bag 92019, Auckland 1142, New Zealand; s.jamieson@auckland.ac.nz; 3 Auckland Cancer Society Research Centre, University of Auckland, Private Bag 92019, Auckland 1142, New Zealand

**Keywords:** anticancer agents, bioorganometallics, metal complexes, pyridinecarbothioamide, metallodrugs, rhodium, iridium

## Abstract

Ru^II^(cym)Cl (cym = η^6^-*p*-cymene) complexes of pyridinecarbothioamides have shown potential for development as orally active anticancer metallodrugs, underlined by their high selectivity towards plectin as the molecular target. In order to investigate the impact of the metal center on the anticancer activity and their physicochemical properties, the Os(cym), Rh- and Ir(Cp*) (Cp* = pentamethylcyclopentadienyl) analogues of the most promising and orally active compound plecstatin **2** were prepared and characterized by spectroscopic techniques and X-ray diffraction analysis. Dissolution in aqueous medium results in quick ligand exchange reactions; however, over time no further changes in the ^1^H NMR spectra were observed. The Rh- and Ir(Cp*) complexes were investigated for their reactions with amino acids, and while they reacted with Cys, no reaction with His was observed. Studies on the in vitro anticancer activity identified the Ru derivatives as the most potent, independent of their halido leaving group, while the Rh derivative was more active than the Ir analogue. This demonstrates that the metal center has a significant impact on the anticancer activity of the compound class.

## 1. Introduction

In antineoplastic drug development, metal complexes have attracted attention due to their unique properties such as structural 3D arrangement, interesting photophysical properties and their ability to form specific interactions with biomolecules [1,2,3]. Platinum-based anticancer drugs are used in a wide number of cancer treatments but their success has been marred due to adverse side effects and intrinsic and developed resistance against these compounds [4,5]. Therefore, there is interest in exploring the tumor-inhibiting properties of other metal-based compounds [6]. Among the non-platinum metallodrugs, ruthenium complexes are particularly promising due to their general low toxicity [7,8,9]. The ruthenium complexes NAMI-A, KP1019 and IT-139 (also known as BOLD-100, KP1339, NKP-1339) have also entered clinical trials [8,10,11]. The discovery of Ru-based RAPTA-C [Ru(cym)(pta)Cl_2_] [cym = η^6^-*p*-cymene, pta = 1,3,5-triaza-7-phoshatricyclo[3.3.1.1]decane) and RAED-C [Ru(cym)(en)Cl]PF_6_ (en = ethylene-1,2-diamine) with contrasting biological activity and different modes of action has fueled research into half-sandwich Ru^II^(η^6^-arene) complexes [12,13,14,15,16]. The arene ligand employed not only stabilizes the oxidation state but also modulates the lipophilic/hydrophilic character of the metal compounds [12]. The antiproliferative activity of Ru(arene) complexes is often dictated by the coordinating ligands or their substituents [17], such as ethacrynic acid [18], chlorambucil [19], quinolines [20,21,22], quinolones [23], lapachol [24], flavonols [25,26], and oxicam [27,28,29] derivatives.

In addition to modifying the ligands, it is also possible to fine-tune the chemical and biological properties of metallodrugs by changing the metal center. Therefore, osmium analogues of many recently reported organoruthenium compounds were tested for their potential as anticancer agents [30]. Osmium complexes have slower ligand exchange reactions compared to their Ru counterparts [31,32]. In addition, half-sandwich rhodium and iridium compounds have emerged as promising chemotherapeutic agents [33].

Pyridine-2-carbothioamides (PCAs) are bioactive *N,S*-bidentate ligands and their Ru^II^ and Os^II^ complexes showed potent cytotoxicity [34,35] along with selective binding to plectin and in vivo activity [36]. Based on preliminary structure-activity relationships, the most active Ru(cym)Cl complex of *p*-fluoro derivative **2** (plecstatin-1) provided a starting point to expand on the compound series by systematic variation of the metal center as well as of the labile halido ligand and to study the impact on the in vitro anticancer activity.

## 2. Results and Discussion

**Scheme 1:**
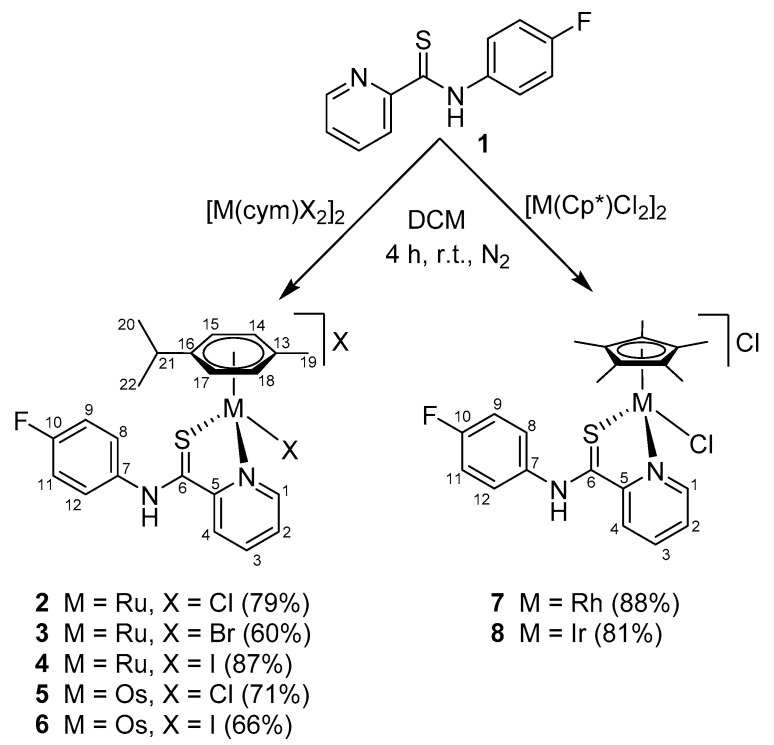
Synthetic route for conversion of *N*-(4-fluorophenyl)pyridine-2-carbothioamide **1** to its organometallic Ru^II^, Os^II^, Rh^III^ and Ir^III^ complexes (**2–8**) along with the NMR spectroscopy numbering scheme.

Careful modifications at the phenyl ring of PCAs and their conversion to Ru(cym)Cl and Os(cym)Cl complexes led to the identification of potent antiproliferative agents [34,35,37]. In order to investigate the effect of different metal ions and halido ligands on the biological properties of PCA-based organometallics, we expanded on the compound class bearing the PCA ligand that resulted in the most cytotoxic Ru(cym)Cl complexes, i.e., 4-fluoro-substituted PCA **1** [34,35]. *N*-(4-Fluorophenyl)pyridine-2-carbothioamide **1** was prepared according to a reported method [34]. It was converted into metal complexes **2**–**8** by reaction with the dimeric precursors [M(π-bound ligand)X_2_]_2_ (M = Ru^II^, Os^II^, Rh^III^, Ir^III^; π-bound ligand = cym, Cp*; X = Cl, Br, I; Scheme 1) for 4 h at 40 °C [35], while **2** and **5** were reported earlier [34]. The novel compounds **3**, **4** and **6**–**8** were isolated in 60–88% yield.

The organometallic compounds were characterized by NMR spectroscopy, ESI-MS, elemental and single crystal X-ray diffraction analyses. The ^1^H NMR spectra of the metal complexes showed that coordination of the metal center had the strongest impact on the chemical shift of H1 which shifted downfield by *ca.*1 ppm compared to **1**. For the *p*-cymene ligand, the aromatic ring protons were observed as four doublets in the range of 5.6–6.2 ppm, while in case of the Cp* complexes **7** and **8**, the Me_Cp*_ protons appeared as a singlet at 1.70 ppm. The ^13^C{^1^H} NMR spectra of the complexes featured the expected peaks, however, the quaternary carbon atoms could not always be detected despite increasing the measurement time. The nature of the complexes was confirmed by ESI-mass spectrometry in positive ion mode. The spectra featured peaks at *m/z* values assignable to the [M − X]^+^ ions while the base peak was attributed to the [M − 2X − H]^+^ ions. A similar ionization behavior in ESI-MS was observed for other PCA–metal complexes [34,35,37,38]. The experimental *m*/*z* values as well as the isotopic distribution pattern closely resembled the calculated values. Furthermore, the formation and purity of the complexes was confirmed by elemental analysis, which gave data close to the theoretical values. In line with signals assignable to *n*-hexane and THF in the ^1^H NMR spectra, residual amounts of the respective solvents were used to calculate the elemental analysis values of **7** and **8**.

**Figure 1:**
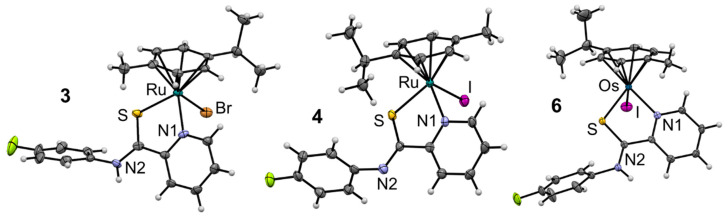
Molecular structures of metal complexes **3**, **4**, and **6** with the thermal ellipsoid drawn at 50% probability level. Solvents and counterions were omitted for clarity.

The molecular structures of complexes **3**, **4**, and **6** were determined by single crystal X-ray diffraction analysis (for crystallographic data and structural refinement parameters see Supplementary Materials, Table S1), and selected bond lengths and bond angles are given in Table 1. Single crystals of **3** were obtained by slow evaporation of a saturated solution in methanol and ethyl acetate, the latter was found to co-crystallize with the complex, while **4** and **6** crystallized from methanolic solutions by slow diffusion of diethyl ether. The molecular structures displayed the expected piano-stool geometry (Figure 1) with PCA **1** coordinating to the metal centers via the pyridine nitrogen and carbothioamide sulfur atoms, forming five-membered rings. Interestingly, complex **4** features a thiolate coordinated to the metal center with N2 being deprotonated, while in case of **3** and **6** the coordinating group is a thioamide. This is also reflected in the charge of the complexes with **3** and **6** being complex cations whereas **4** is charge neutral. The N2 protonation state has a significant impact on the bond lengths observed with the Ru–S bond and C6–S in **4** being longer than in the analogous cationic complexes (Table 1). Accordingly, the C6–N2 bond in **4** has higher single bond character than the analogous bonds in **3** and **6**. Furthermore, the Ru–I bond in **4** is significantly longer than the Os–I in **6**, while overall the bond angles were very similar for all three complexes. We investigated the deprotonation process by density functional theory (DFT) calculations in methanol and found that the deprotonated form was about 30 kcal mol^−1^ energetically less favorable than the protonated complex, independent of the metal center.

**Figure 2:**
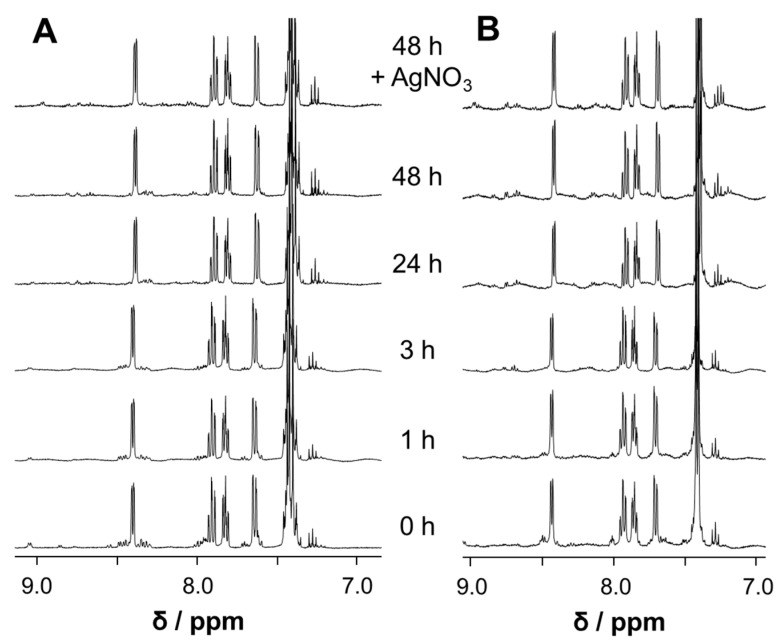
The stability of complexes **7** (**A**) and **8** (**B**) in D_2_O investigated by ^1^H NMR spectroscopy over a period of 48 h. After 48 h, AgNO_3_ was added to the sample and another spectrum was recorded.

The stability of complexes **7** and **8** was investigated in an analogous manner as reported earlier for the Ru and Os congeners [34]. ^1^H NMR spectra recorded over a period of 48 h in D_2_O and addition of AgNO_3_ to abstract the coordinating halido ligands demonstrated that quick ligand exchange reactions occured and the formed species were found to be stable over the duration of the experiment (Figure 2).

**Figure 3:**
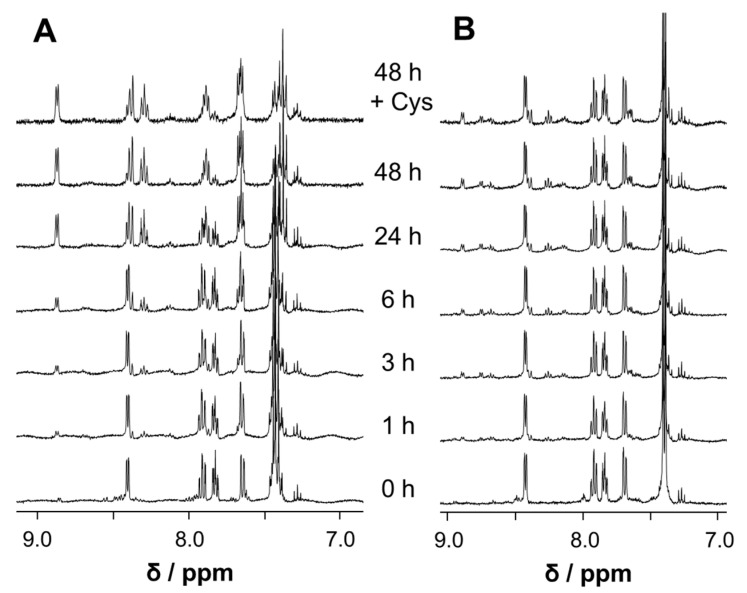
The reactions of **7** (**A**) and **8** (**B**) with Cys (1:1) in D_2_O over a period of 48 h by ^1^H NMR spectroscopy. After 48 h, another equivalent of Cys was added to either reaction mixture and analyzed again by ^1^H NMR spectroscopy.

We have shown earlier that **2** can react with proteins [34]. Here, we investigated the reactions of the Rh and Ir complexes **7** and **8** with the amino acids l-cysteine (Cys) and l-histidine (His) at equimolar ratios by ^1^H NMR spectroscopy in D_2_O. While no binding with His was observed, both compounds reacted with Cys, resulting in additional peaks in the ^1^H NMR spectra (Figure 3). Not surprisingly, the reaction with the more kinetically inert Ir derivative **8** was found to proceed more slowly than with Rh complex **7**. However, in both cases significant changes in the aromatic regions of the ^1^H NMR spectra were visible, in particular new peaks emerging which resonated at lower fields than the pyridine H1 protons, while others overlapped with peaks of the original complexes (Figure 3).

PCAs, being gastric mucosal protectants, are known to have low toxicity in vivo, suggesting better tolerability [39], despite showing IC_50_ values in the μM range (Table 2) [35]. Coordination to organometallic moieties based on Ru and Os resulted in potent cytotoxins with similar activity to the ligands [34,35] and promising in vivo activity [36]. The preparation of **3**, **4** and **6**–**8** allowed us to explore further factors as part of structure-activity relationship determinations. Therefore, the compounds were compared to the Ru(cym)Cl **2** and Os(cym)Cl **5** derivatives in terms of their antiproliferative activity against the human cancer cell lines HCT116, NCI-H460, SiHa and SW480 (Table 2). In general, the complexes demonstrated anticancer activity with IC_50_ values in the low μM range. Comparing the IC_50_ value of the 4-fluoro substituted PCA **1**, it is obvious that it has a major impact on the biological activity of the complexes. However, within the complexes significant differences were observed. Examination of the data for the Ru complexes **2**–**4** with the labile chlorido, bromido and iodido ligands showed only minor differences in their cytotoxicity, which can be explained by the previously reported rapid halido aqua exchange for **2** and related complexes in aqueous solution, independent of the nature of the halide [34,37]. The same argument can be made for the Os compounds **5** and **6** which gave very similar IC_50_ values, although slightly lower than their Ru congeners. Similarly, the Rh and Ir compound pair **7** and **8** indicated slightly higher activity for the more kinetically labile Rh congener, which supports to some extent the notion that direct coordination of the metal center to the biomolecular target, i.e., plectin, is critical for the anticancer activity [36]. The importance of the chlorido/aqua exchange reaction and its influence on the target selectivity further supports the latter observation [40].

## 3. Materials and Methods

All air- and moisture-sensitive reactions were carried out under N_2_ atmosphere using standard Schlenk techniques. Chemicals obtained from commercial suppliers were used as received and were of analytical grade. Tetrahydrofuran (THF), dichloromethane (DCM), and diethyl ether (Et_2_O) were dried through a solvent purification system (LC Technology Solutions Inc., Salisbury, MA, USA, SP-1 solvent purifier), degassed under a N_2_ flow, and then stored in a Schlenk flask. Ethanol (EtOH) and methanol (MeOH) were dried over activated molecular sieves (3 Å) in Erlenmeyer flasks for two days prior to use.

4-Fluoroaniline, α-terpinene, 2-picoline, and Na_2_S·9H_2_O were purchased from Merck, sulfur and osmium tetroxide (98%) were purchased from Sigma-Aldrich, Auckland, New Zealand. Ruthenium(III) chloride hydrate (99%), iridium(III) chloride hydrate and rhodium(III) chloride hydrate were from Precious Metals Online (Wollongong, Australia).

*N*-(4-Fluorophenyl)pyridine-2-carbothioamide **1**, [chlorido(η^6^-*p*-cymene)(*N*-(4-fluorophenyl)pyridine-2-carbothioamide)ruthenium(II)] chloride **2** and [chlorido(η^6^-*p*-cymene)(*N*-(4-fluorophenyl)pyridine-2-carbothioamide)osmium(II)] chloride **5** were synthesized by adapting reported procedures [34]. The dimeric precursors bis[dichlorido(η^6^-*p*-cymene)ruthenium(II)] [41], bis[dichlorido(η^6^-*p*-cymene)osmium(II)] [42], bis[dichlorido(η^5^-pentamethylcyclopentadienyl)rhodium(III)] [43] and bis[dichlorido(η^5^-pentamethylcyclopentadienyl)iridium(III)] [44] were synthesized by following literature procedures.

### 3.1. Physical Measurements

^1^H, ^13^C{^1^H} and 2D (COSY, HSQC, HMBC) NMR spectra were recorded in DMSO-*d*_6_, MeOH-*d*_4_ or CDCl_3_ on a Bruker Avance AVIII400 MHz NMR spectrometer (Billerica, MA, USA) at 400.13 (^1^H) or 100.61 MHz (^13^C{^1^H}) and ambient temperature. Chemical shifts are reported versus SiMe_4_ and are reported by reference to the residual solvent peaks.

The mass spectra were recorded on a Bruker micrOTOF-QII (Bremen, Germany) mass spectrometer in positive electrospray ionization (ESI) mode. Elemental analyses were carried out on an Exeter Analytical Inc-CE-440 Elemental Analyser (Exeter Analytical Limited, Coventry, United Kingdom). X-ray diffraction measurements of single crystals of **3**, **4** and **6** were performed on a Bruker SMART APEX2 (Bruker Coorporation, Karlsruhe, Germany & Madison, WI, USA) diffractometer with a CCD area detector using graphite monochromated Mo-Kα radiation (λ = 0.71073 Å). The molecular structures were solved and refined with the SHELXL-2016 [45] (Sheldrick, GM, University of Göttingen, Germany) and Olex2 [46,47] (OlexSys GmbH, Regensburg, Germany) program packages. The molecular structures were visualized using Mercury 2020.3 (The Cambridge Crystallographic Data Centre, Cambridge, UK).

### 3.2. Syntheses

*General procedure*. The complexes were synthesized by combining DCM solutions of carbothioamide **1** (1 eq.) and the respective dimeric precursors [M(π-bound ligand)X_2_]_2_ (0.5 eq.). The reaction mixture was stirred for 4 h at room temperature under nitrogen atmosphere. The solvent was concentrated in vacuo to ca. 5 mL and *n*-hexane was added to initiate precipitation in the fridge. The solvent was decanted and subsequent drying in vacuo yielded analytically pure solid product.

*[Bromido(η^6^-p-cymene)(N-(4-fluorophenyl)pyridine-2-carbothioamide)ruthenium(II)] bromide* (**3**). Compound **3** was synthesized following the general procedure using *N*-(4-fluorophenyl)-2-pyridinecarbothioamide **1** (75 mg, 0.32 mmol) and [Ru(cym)Br_2_]_2_ (128 mg, 0.13 mmol). Yield: 121 mg (60%), red solid. Elemental analysis found: C, 42.34; H, 3.88; N, 4.54, calculated for C_22_H_23_Br_2_FN_2_RuS: C, 42.12; H, 3.70; N, 4.47. ^1^H NMR (400.13 MHz, MeOD-*d_4_*, 25 °C): δ = 9.67 (d, ^3^*J* = 6 Hz, 1H, H-1), 8.44 (d, ^3^*J* = 8 Hz, 1H, H-4), 8.29 (td, ^3^*J* = 8 Hz, ^4^*J* = 1 Hz, 1H, H-3), 7.83 (td, ^3^*J* = 7 Hz, ^4^*J* = 1 Hz, 1H, H-2), 7.64–7.68 (m, 2H, H-9/H-11), 7.35 (t, ^3^*J* = 8 Hz, 2H, H-8/H-12), 6.04 (d, ^3^*J* = 6 Hz, 1H, H-15), 5.92 (d, ^3^*J* = 6 Hz, 1H, H-17), 5.89 (d, ^3^*J* = 6 Hz, 1H, H-18), 5.68 (d, ^3^*J* = 6 Hz, 1H, H-14), 2.80 (sept, ^3^*J* = 6 Hz, 1H, H-21), 2.28 (s, 3H, H-19), 1.21 (d, ^3^*J* = 6 Hz, 3H, H-20), 1.15 (d, ^3^*J* = 7 Hz, 3H, H-22) ppm. ^13^C{^1^H} NMR (100.61 MHz, MeOD-*d_4_*, 25 °C): δ = 194.1 (C-6), 165.0 (C-10), 162.5 (C-5), 160.8 (C-1), 154.7 (C-7), 141.0 (C-3), 130.7 (C-2), 128.9 (C-9), 125.8 (C-11), 125.0 (C-4), 117.9 (C-8), 117.7 (C-12), 108.2 (C-16), 105.0 (C-13), 89.3 (C-15), 89.2 (C-17), 86.7 (C-18), 85.8 (C-14), 32.6 (C-21), 22.9 (C-20), 21.9 (C-22), 19.3 (C-19) ppm. MS (ESI^+^): *m*/*z* 467.0531 [**3** − 2Br − H]^+^ (m_ex_ = 467.0526).

*[Iodido(η^6^-p-cymene)(N-(4-fluorophenyl)pyridine-2-carbothioamide)ruthenium(II)] iodide* (**4**). Compound **4** was synthesized following the general procedure using *N*-(4-fluorophenyl)pyridine-2-carbothioamide **1** (65 mg, 0.28 mmol) and [Ru(cym)I_2_]_2_ (137 mg, 0.14mmol). Yield: 179 mg (87%), red solid. Elemental analysis found: C, 36.87; H, 3.20; N, 3.66, calculated for C_22_H_23_FI_2_N_2_RuS: C, 36.63; H, 3.21; N, 3.88. ^1^H NMR (400.13 MHz, MeOD-*d_4_*, 25 °C): δ = 9.64 (d, ^3^*J* = 6 Hz, 1H, H-1), 8.42 (d, ^3^*J* = 9 Hz, 1H, H-4), 8.26 (td, ^3^*J* = 8 Hz, ^4^*J* = 1 Hz, 1H, H-3), 7.76 (td, ^3^*J* = 7 Hz, ^4^*J* = 1 Hz, 1H, H-2), 7.63–7.68 (m, 2H, H-9/H-11), 7.35 (t, ^3^*J* = 8 Hz, 2H, H-8/H-12), 6.03 (d, ^3^*J* = 6 Hz, 1H, H-15), 5.89 (d, ^3^*J* = 6 Hz, 1H, H-17), 5.86 (d, ^3^*J* = 6 Hz, 1H, H-18), 5.71 (d, ^3^*J* = 6 Hz, 1H, H-14), 2.89 (sept, ^3^*J* = 6 Hz, 1H, H-21), 2.37 (s, 3H, H-19), 1.21 (d, ^3^*J* = 7 Hz, 3H, H-20), 1.17 (d, ^3^*J* = 7 Hz, 3H, H-22) ppm. ^13^C{^1^H} NMR (100.61 MHz, MeOD-*d_4_*, 25 °C): δ = 164.9 (C-10), 162.4 (C-5), 154.9 (C-7), 140.7 (C-3), 130.2 (C-2), 128.9 (C-9), 125.8 (C-11), 125.1 (C-4), 109.3 (C-16), 104.7 (C-13), 89.5 (C-15), 89.1 (C-17), 86.9 (C-18), 86.8 (C-14), 33.0 (C-21), 23.0 (C-20), 22.1 (C-22), 20.1 (C-19) ppm. MS (ESI^+^): *m*/*z* 467.0531 [**4** − 2I − H]^+^ (m_ex_ = 467.0538).

*[Iodido(η^6^-p-cymene)(N-(4-fluorophenyl)pyridine-2-carbothioamide)osmium(II)] iodide* (**6**). DCM solutions of *N*-(4-fluorophenyl)pyridine-2-carbothioamide **1** (55 mg, 0.24 mmol) and [Os(cym)I_2_]_2_ (137 mg, 0.12 mmol) were combined and stirred for 4 h at room temperature under nitrogen atmosphere. After completion of the reaction, the solid product was filtered followed by washing with dichloromethane (2 × 10 mL) and tetrahydrofuran (10 mL) and drying in vacuum. Yield: 130 mg (66%), black solid. Elemental analysis found: C, 32.82, H, 2.86, N, 3.37, S, 3.96, calculated for C_22_H_23_FI_2_N_2_OsS: C, 32.60; H, 2.86; N, 3.46 S, 3.96. ^1^H NMR (400.13 MHz, MeOD-*d_4_*, 25 °C): δ = 9.60 (d, ^3^*J* = 6 Hz, 1H, H-1), 8.47 (d, ^3^*J* = 9 Hz, 1H, H-4), 8.22 (td, ^3^*J* = 7 Hz, ^4^*J* = 2 Hz, 1H, H-3), 7.71 (td, ^3^*J* = 7 Hz, ^4^*J* = 1 Hz, 1H, H-2), 7.62–7.67 (m, 2H, H-9/H-11), 7.35 (t, ^3^*J* = 8 Hz, 2H, H-8/H-12), 6.18 (d, ^3^*J* = 6 Hz, 1H, H-15), 6.06 (d, ^3^*J* = 6 Hz, 1H, H-17), 6.03 (d, ^3^*J* = 6 Hz, 1H, H-18), 5.87 (d, ^3^*J* = 6 Hz, 1H, H-14), 2.78 (sept, ^3^*J* = 6 Hz, 1H, H-21), 2.43 (s, 3H, H-19), 1.20 (d, ^3^*J* = 7 Hz, 3H, H-20), 1.13 (d, ^3^*J* = 7 Hz, 3H, H-22) ppm. ^13^C{^1^H} NMR (100.61 MHz, MeOD-*d_4_*, 25 °C): δ = 164.8 (C-10), 162.6 (C-5), 140.6 (C-3), 131.2 (C-2), 128.9 (C-9), 128.8 (C-11), 125.4 (C-4), 117.9 (C-8), 117.7 (C-12), 100.5 (C-16), 97.1 (C-13), 81.5 (C-15), 81.5 (C-17), 78.9 (C-18), 78.1 (C-14), 32.9 (C-21), 23.2 (C-20), 22.1 (C-22), 20.0 (C-19) ppm. MS (ESI^+^): *m*/*z* 557.1103 [**6** − 2I − H]^+^ (m_ex_ = 557.1115).

*[Chlorido(η^5^-pentamethylcyclopentadienyl)(N-(4-fluorophenyl)pyridine-2-carbothioamide)rhodium(III)] chloride* (**7**). Compound **7** was synthesized following the general procedure using *N*-(4-fluorophenyl)pyridine-2-carbothioamide **1** (130 mg, 0.56 mmol) and [Rh(Cp*)Cl_2_]_2_ (173 mg, 0.28 mmol). Yield: 268 mg (88%), orange solid. Elemental analysis found: C, 50.80; H, 4.70; N, 5.35, calculated for C_22_H_24_Cl_2_FN_2_RhS·0.3C_6_H_14_ C, 50.40; H, 5.01; N,4.94. ^1^H NMR 400.13 MHz, CDCl_3_, 25 °C): δ = δ = 9.28 (d, ^3^*J* = 7 Hz, 1H, H-1), 8.76 (d, ^3^*J* = 5 Hz, 1H, H-4), 8.13 (t, ^3^*J* = 8 Hz, 1H, H-3), 7.77 (t, ^3^*J* = 7, 2H, H-9/H-11), 7.62 (t, ^3^*J* = 7 Hz, 1H, H-2), 7.15 (t, ^3^*J* = 9 Hz, 2H, H-8/H-12), 1.68 (s, 15H, CH_3_-Cp*) ppm. ^13^C{^1^H} NMR (100.61 MHz, CDCl_3_, 25 °C): δ = 162.8 (C-10), 160.4 (C-5),153.8 (C-1), 140.1 (C-3), 128.8 (C-9/C-11), 127.1 (C-2), 126.6 (C-4), 116.2 (C-8), 116.0 (C-12), 97.5 (^2^*J_Rh-C_* = 16 Hz, Rh, Cp*-C), 9.1 (Cp*-CH_3_) ppm. MS (ESI^+^): *m*/*z* 469.0621 [**7** − 2Cl − H]^+^ (m_ex_ = 469.0614).

*[Chlorido(η^5^-pentamethylcyclopentadienyl)(N-(4-fluorophenyl)pyridine-2-carbothioamide)iridium(III)] chloride* (**8**). Compound **8** was synthesized following the general procedure using *N*-(4-fluorophenyl)pyridine-2-carbothioamide **1** (100 mg, 0.40 mmol) and [Ir(Cp*)Cl_2_]_2_ (123 mg, 0.20 mmol). Yield: 176 mg (81%), red solid. Elemental analysis found: C, 42.71; H, 3.80; N, 4.31, calculated for C_22_H_24_Cl_2_FIrN_2_S·0.1C_4_H_8_O·0.1C_6_H_14_: C, 42.73; H, 4.09; N, 4.33. ^1^H NMR (400.13 MHz, CDCl_3_, 25 °C): δ = 9.30 (d, ^3^*J* = 6 Hz, 1H, H-1), 8.74 (d, ^3^*J* = 5 Hz, 1H, H-4), 8.08 (t, ^3^*J* = 7 Hz, 1H, H-3), 7.74 (t, ^3^*J* = 7 H, 2H, H-9/H-11), 7.56 (t, ^3^*J* = 6 Hz, 1H, H-2), 7.15 (t, ^3^*J* = 9 Hz, 2H, H-8/H-12), 1.70 (s, 15H, CH_3_-Cp*) ppm. ^13^C{^1^H} NMR (100.61 MHz, CDCl_3_, 25 °C): δ = 154.5 (C-1), 139.8 (C-3), 129.2 (C-9/C-11), 126.3 (C-2), 116.2 (C-4), 115.9 (C-8/C-12), 90.1 (Cp*-C), 8.8 (Cp*-CH_3_) ppm. MS (ESI^+^): *m*/*z* 559.1195 [**8** − 2Cl − H]^+^ (m_ex_ = 559.1200).

### 3.3. Sulforhodamine B Cytotoxicity Assay

The in vitro cytotoxicity of the compounds was investigated in HCT116, SW480, NCI-H460, and SiHa cells, as described elsewhere [35].

### 3.4. Stability and Amino Acid Reactivity Studies

For the stability studies, **7** and **8** (1–2 mg) were dissolved in D_2_O and ^1^H NMR spectra were recorded over a period of 48 h. After 48 h, equimolar amounts of AgNO_3_ were added and another ^1^H NMR spectrum was recorded.

To investigate the reactions with amino acids, **7** and **8** (1–2 mg) were incubated with His or Cys in D_2_O and ^1^H NMR spectra were recorded over a period of 48 h. After 48 h, equimolar amounts of the respective amino acids were added and another ^1^H NMR spectrum was recorded.

### 3.5. DFT Calculations

GAUSSIAN 09W [48] was used to calculate the optimized ground state structures and frequencies for the different molecules by density functional theory (DFT), as described earlier [49]. The B3LYP-D3 hybrid exchange functional was used as well as a split basis set for C, H, N, S, F and Cl (6-31G(d,p)) and the transition metals iridium, osmium, rhodium, and ruthenium (SDDAll) in vacuum.

## 4. Conclusions

The coordination of PCA ligands to metal ions has resulted in compounds with promising in vitro and in vivo anticancer activity [34,35,36]. Herein, we investigated the impact of the metal center as well as of the labile halido ligand on the stability as well as on the anticancer activity against a small panel of standard cell lines. We prepared the Ru^II^, Os^II^, Rh^III^ and Ir^III^ complexes of *N*-4-fluorophenyl pyridine-2-carbothioamide **1** containing different halido leaving groups (Cl, Br, I) and characterized them spectroscopically and in case of the Ru(cym)Br, Ru(cym)I and Os(cym)I derivatives by X-ray diffraction analysis. Interestingly, the Ru(cym)I crystallized in the deprotonated, neutral form while the other two complexes were found to crystallize as their complex cations, which appear to be prevalent when isolated synthetically and were shown by DFT calculations to be energetically preferred. In vitro anticancer activity studies revealed the highest potency for the Ru complexes, independent of their halido ligand, while all derivatives showed cytotoxic activity in the low μM range of around 10 μM. We associate the similar potency of the Ru complexes to their ligand exchange properties in an aqueous environment, which results in the rapid formation of the respective aqua complexes and suggests that covalent bond formation is essential for the mode of action of the complexes. This is supported by the slightly lower activity of the isostructural Os complexes, a trend also reflected for the Rh and Ir analogs.

## Figures and Tables

**Table 1:** Key bond lengths (Å) and angles (°) for **3**, **4**, and **6**.

Complex	3·EtAc	4	6
Bond length/Å			
M–S	2.3430(11)	2.3671(10)	2.3519(9)
C6–S	1.689(4)	1.731(4)	1.682(4)
C6–N2	1.326(5)	1.285(5)	1.330(4)
M–N1	2.103(3)	2.096(3)	2.099(3)
M–X1	2.5465(6)	2.7331(4)	2.7165(3)
M–cym_centroid_	1.701	1.706	1.690
Bond angle/°			
S–M–N1	81.54(10)	81.55(9)	80.50(8)
N1–M–X1	82.36(10)	84.14(9)	83.56(8)
S–M–X	89.50(3)	89.75(3)	89.74(2)

**Table 2:** IC_50_ values (µM) for complexes **1**–**8** against HCT116 (human colorectal carcinoma), NCI-H460 (human non-small cell lung carcinoma), SiHa (human cervical carcinoma), and SW480 (human colon adenocarcinoma) cancer cells determined with the SRB assay and expressed as mean ± standard error (*n* = 3). Incubation time: 72 h.

Compound	IC_50_ Values (μM)			
	HCT116	NCI-H460	SiHa	SW480
**1** *^a^*	5.7 ± 0.7	7.8 ± 1.8	16 ± 6	9.9 ± 0.7
**2** *^a^*	6.5 ± 0.3	10 ± 2	8.3 ± 0.7	4.3 ± 1.2
**3**	7.7 ± 0.5	6.8 ± 1.0	17 ± 2	7.6 ± 0.7
**4**	7.5 ± 0.3	7.1 ± 0.9	17 ± 1	7.5 ± 0.8
**5**	18 ± 1	24 ± 2	21 ± 3	10 ± 2
**6**	19 ± 1	18 ± 1	31 ± 2	24 ± 1
**7**	11 ± 2	12 ± 2	22 ± 5	8.9 ± 1.1
**8**	15 ± 2	18 ± 4	46 ± 6	24 ± 6

*^a^* taken from ref. [35].

## Data Availability

No new data were created or analyzed in this study.

## Supplementary Materials

The following are available online, ^1^H, ^13^C{^1^H} NMR spectra, data collection parameters for X-ray diffraction analyses, stability in D_2_O and data on the reactions of **7** and **8** with His.

## Sample Availability

Samples of the compounds are not available from the authors.
