# Supplementary material for: Impact of the Metal Center and Leaving Group on the Anticancer Activity of Organometallic Complexes of Pyridine-2-carbothioamide
Source: Molecules. 2021 Feb 5;26(4):833. doi: 10.3390/molecules26040833 (PMC7914729; doi:10.3390/molecules26040833)
Supplement: Supplementary file 1 [file molecules-26-00833-s001.pdf]

Supporting information

to

Impact of the Metal Center and Leaving Group on the Anticancer Activity of Organometallic Complexes of Pyridine-2-carbothioamide

Jahanzaib Arshad [1], Kelvin K.H. Tong [1,2], Sanam Movassaghi[1], Tilo Söhnel [1], Stephen M. F. Jamieson [2,3], Muhammad Hanif [1,2]*, and Christian G. Hartinger [1,2]*

[1] School of Chemical Sciences, University of Auckland, Private Bag 92019, Auckland 1142, New Zealand

[2] Maurice Wilkins Centre, University of Auckland, Private Bag 92019, Auckland, 1142, New Zealand

[3] Auckland Cancer Society Research Centre, University of Auckland, Private Bag 92019, Auckland 1142, New Zealand

* Correspondence: c.hartinger@auckland.ac.nz (C.G. H.); m.hanif@auckland.ac.nz (M.H.); Tel.: +64-9-3737599 83220 (C.G.H.); web: http://hartinger.auckland.ac.nz/

Table of Contents

X-ray diffraction measurement parameters

Stability and reactivity investigations

NMR spectra

Table S1. X-ray diffraction parameters for the analysis of single crystals of **3**, **4**, and **6**.

	**3**·$C_4H_8O_2$	**4**	**6**
CCDC	2050471	2050472	2050473
Formula	$C_{22}H_{23}Br_2FN_2RuS \cdot C_4H_8O_2$	$C_{22}H_{23}FIN_2RuS$	$C_{22}H_{23}FI_2N_2OsS$
Molecular weight / g mol^{-1}	715.48	594.48	810.48
Crystal description	red block	dark red block	dark red block
Crystal size / mm × mm × mm	0.38 × 0.10 × 0.05	0.28 × 0.28 × 0.22	0.35 × 0.34 × 0.10
Wavelength / Å	0.71073	0.71073	0.71073
Temperature / K	99(2)	99(2)	99(4)
Crystal system	monoclinic	monoclinic	monoclinic
Space group	$P2_1/n$	$P2_1/n$	$P2_1/c$
a / Å	14.1004(4)	6.4580(2)	15.2088(12)
b / Å	8.9443(3)	17.6353(6)	11.3216(9)
c / Å	22.6996(6)	19.1159(7)	13.9552(11)
β / °	91.8909(18)	96.899(2)	94.179(4)
Volume / $Å^3$	2861.27(15)	2161.32(13)	2396.5(3)
Z	4	4	4
ρ_{calc} / g cm^{-3}	1.661	1.824	2.246
μ / mm^{-1}	3.445	2.269	8.003
F(000)	1424.0	1160.0	1504.0
2Θ range for data collection / °	3.352 to 55.676	5.094 to 55.638	5.252 to 55.728
Index ranges	$-18 \leq h \leq 17$<br>$-11 \leq k \leq 11$<br>$-29 \leq l \leq 29$	$-8 \leq h \leq 8$<br>$-23 \leq k \leq 22$<br>$-24 \leq l \leq 25$	$-19 \leq h \leq 19$<br>$-12 \leq k \leq 14$<br>$-17 \leq l \leq 18$
Reflections collected	32770	25783	26570
Independent reflections	6780<br>[R_{int} = 0.0968,<br>R_{sigma} = 0.0869]	5098<br>[R_{int} = 0.0493,<br>R_{sigma} = 0.0456]	5660<br>[R_{int} = 0.0541,<br>R_{sigma} = 0.0413]
Data/restraints/parameters	6780/0/324	5098/0/256	5660/0/269
Goodness-of-fit on F^2	1.013	1.039	1.084
Final R indexes [I ≥ 2σ (I)]	R_1 = 0.0436,<br>wR_2 = 0.0811	R_1 = 0.0383,<br>wR_2 = 0.0846	R_1 = 0.0261,<br>wR_2 = 0.0655
Final R indexes [all data]	R_1 = 0.0825,<br>wR_2 = 0.0933	R_1 = 0.0534,<br>wR_2 = 0.0912	R_1 = 0.0284,<br>wR_2 = 0.0668
Largest diff. peak/hole / e $Å^{-3}$	0.97/-0.70	1.61/-0.65	1.52/-1.46

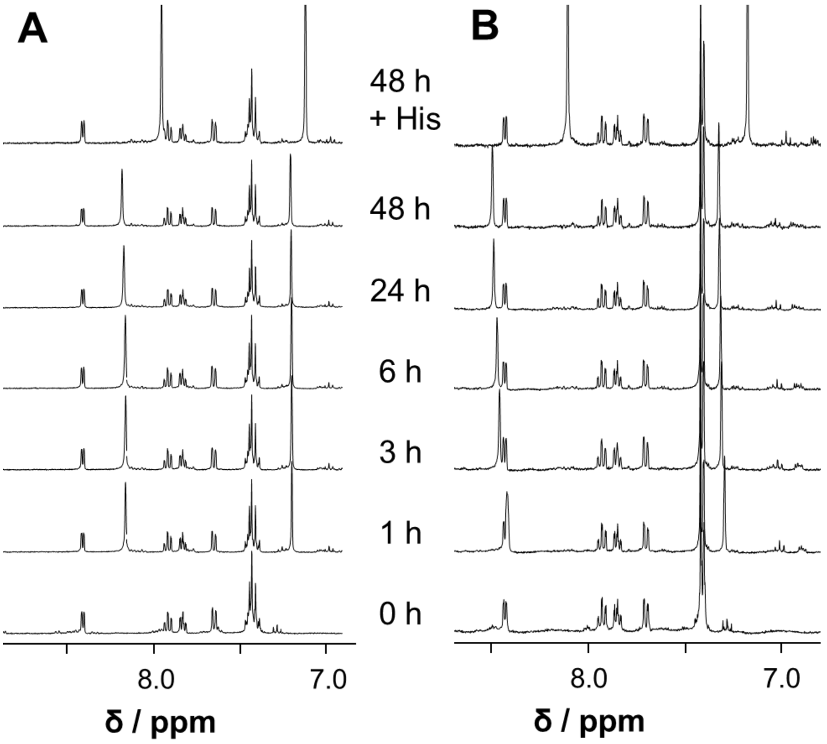

Figure S1. The reactions of **7** (A) and **8** (B) with His (1 : 1) in D_2O over a period of 48 h by ^{1}H NMR spectroscopy. After 48 h, another equivalent of His was added.

NMR spectra

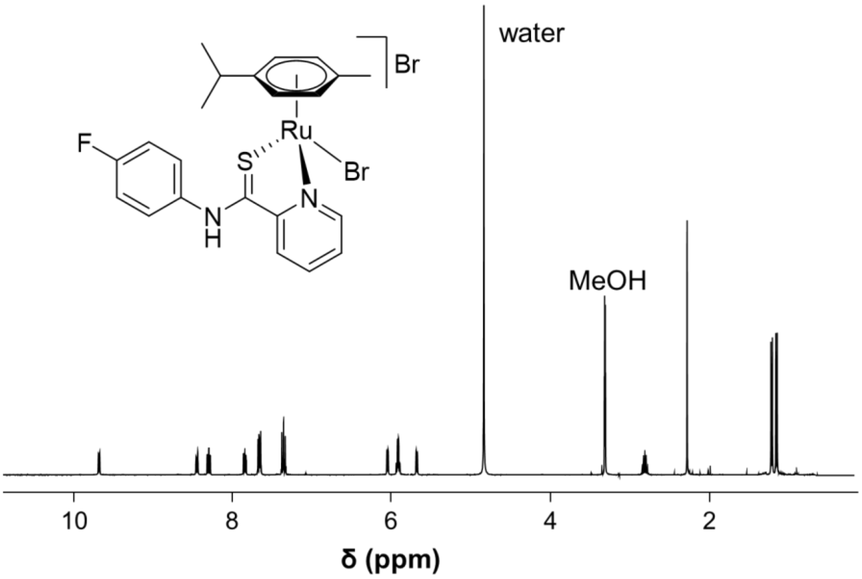

Figure S2. ^{1}H NMR spectrum of **3** in d_4-MeOD.

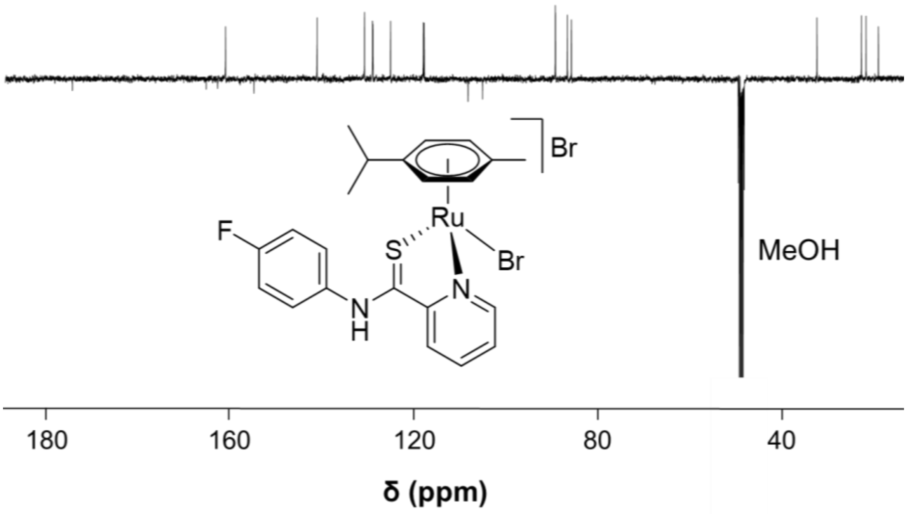

Figure S3. $^{13}C\{^{1}H\}$ NMR spectrum of **3** in d_4-MeOD.

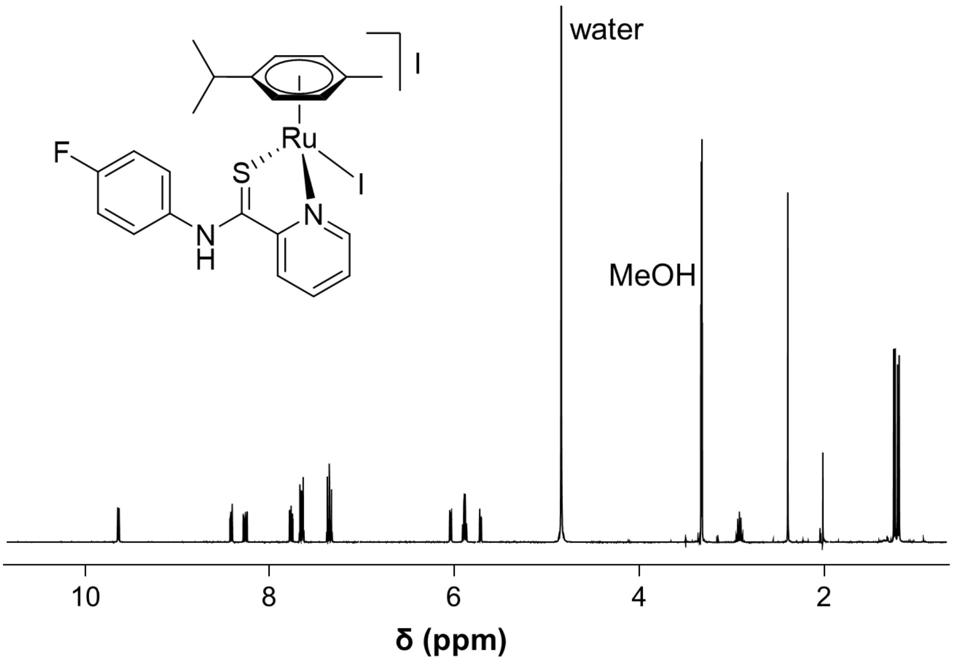

Figure S4. ^{1}H NMR spectrum of **4** in d_4-MeOD.

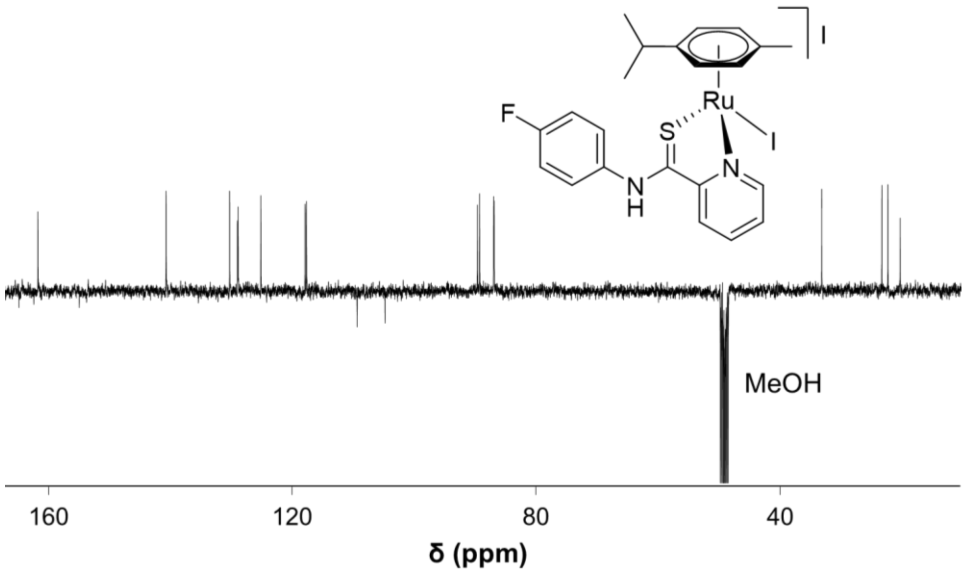

Figure S5. $^{13}C\{^{1}H\}$ NMR spectrum of **4** in d_4-MeOD.

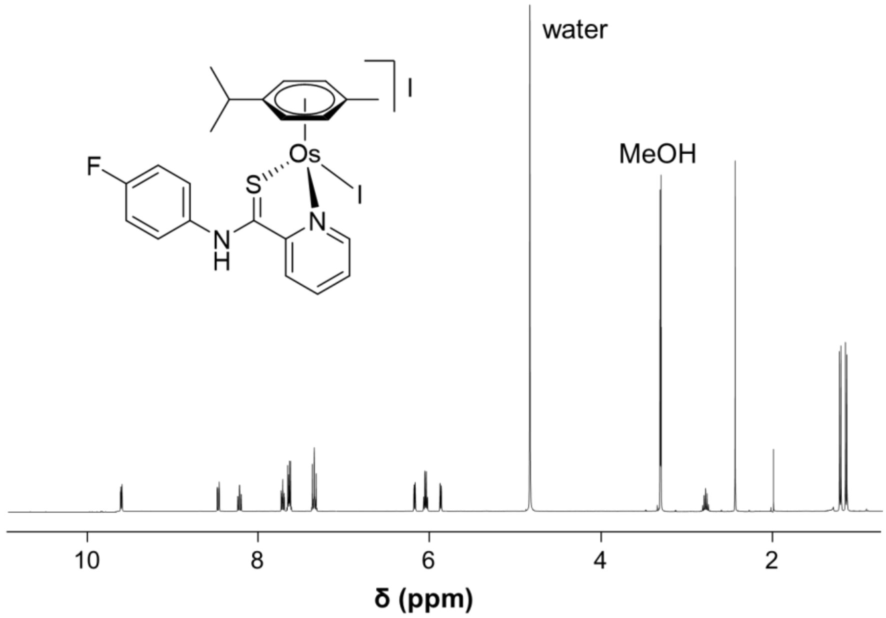

Figure S6. ^{1}H NMR spectrum of **6** in d_4-MeOD.

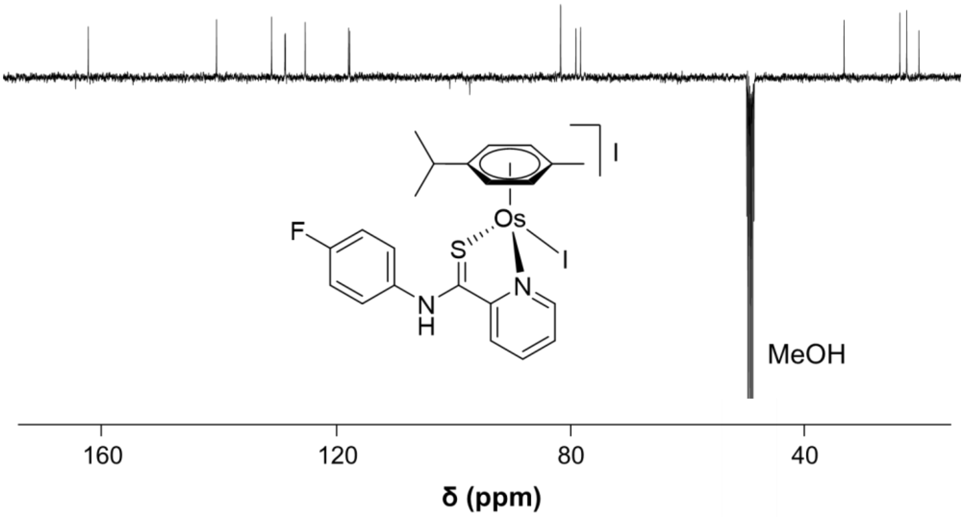

Figure S7. $^{13}C\{^{1}H\}$ NMR spectrum of **6** in d_4-MeOD.

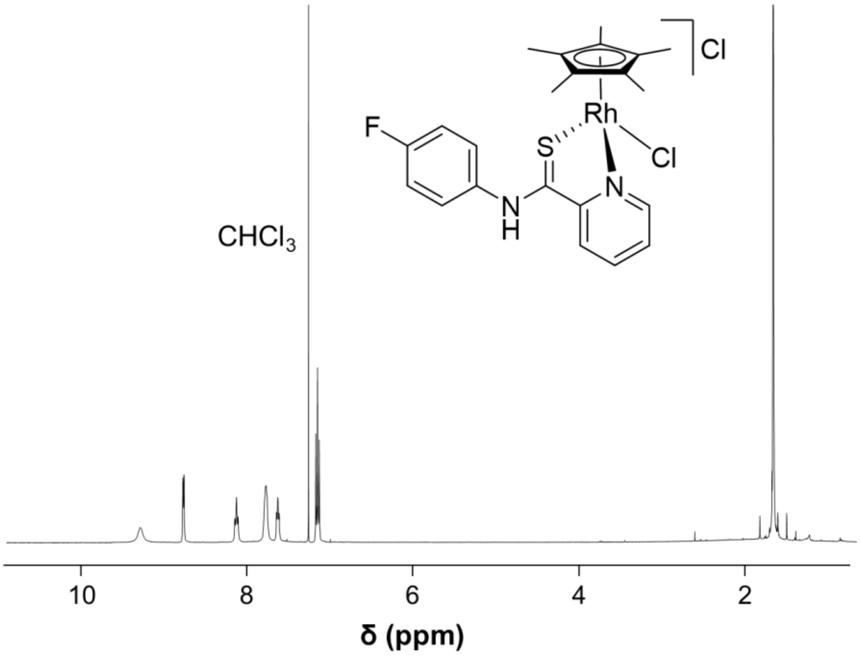

Figure S8. ^{1}H NMR spectrum of **7** in $CDCl_3$.

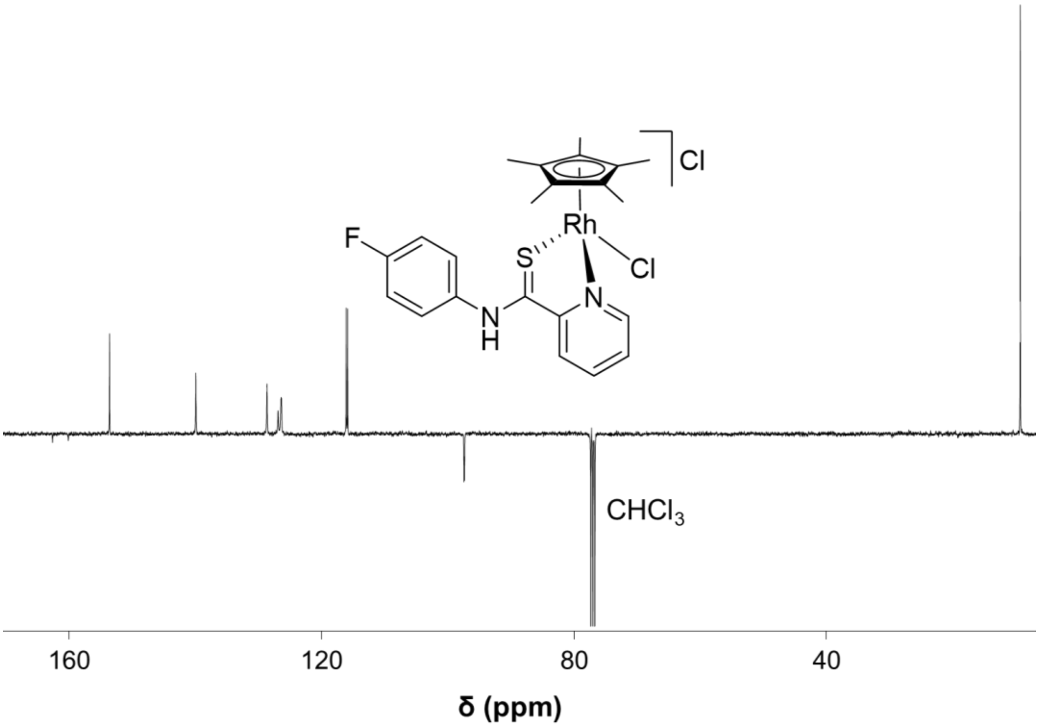

Figure S9. $^{13}C\{^{1}H\}$ NMR spectrum of **7** in *d*$_4$-MeOD.

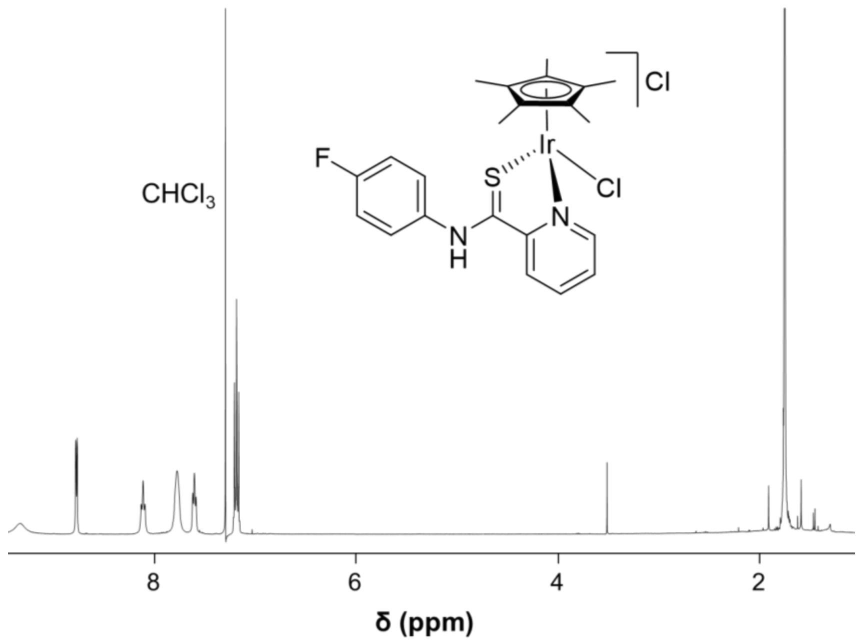

Figure S10. ^{1}H NMR spectrum of **8** in $CDCl_3$.

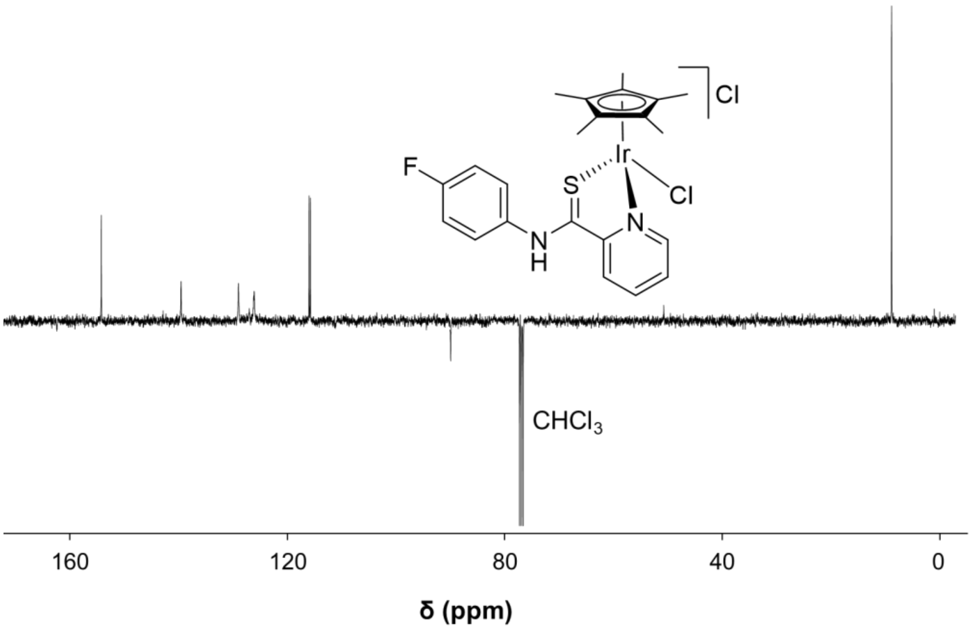

Figure S11. $^{13}C\{^{1}H\}$ NMR spectrum of **8** in $CDCl_3$.
